# Radiologic Differentiation between Granulomatosis with Polyangiitis and Its Mimics Involving the Skull Base in Humans Using High-Resolution Magnetic Resonance Imaging

**DOI:** 10.3390/diagnostics11112162

**Published:** 2021-11-22

**Authors:** Boeun Lee, Yun Jung Bae, Byung Se Choi, Byung Yoon Choi, Se Jin Cho, Hyojin Kim, Jae Hyoung Kim

**Affiliations:** 1 Department of Radiology, College of Medicine, Ewha Womans University, Ewha Womans University Seoul Hospital, 260 Gonghang-daero, Gangseo-gu, Seoul 07804, Korea; boeunlee1117@naver.com; 2 Department of Radiology, Seoul National University Bundang Hospital, 82 Gumi-ro 173beon-gil, Bundang-gu, Seongnam 13620, Korea; sejinchorad@gmail.com (S.J.C.); jaehkim@snu.ac.kr (J.H.K.); 3 Department of Otolaryngology, Seoul National University Bundang Hospital, 82 Gumi-ro 173beon-gil, Bundang-gu, Seongnam 13620, Korea; choiby2010@gmail.com; 4 Department of Pathology, Seoul National University Bundang Hospital, 82 Gumi-ro 173beon-gil, Bundang-gu, Seongnam 13620, Korea; hyojinkim7137@gmail.com

**Keywords:** granulomatosis with polyangiitis, skull base osteomyelitis, nasopharyngeal carcinoma, magnetic resonance imaging

## Abstract

Granulomatosis with polyangiitis (GPA) can involve the skull base or the Eustachian tubes. GPA is diagnosed on the basis of clinical manifestations and serological tests, although it is challenging to discriminate GPA from infectious processes driving skull base osteomyelitis (SBO) and malignant processes such as nasopharyngeal carcinoma (NPC). Moreover, current serological tests have a low sensitivity and cannot distinguish GPA from these other conditions. We hypothesized that certain MRI characteristics would differ significantly among conditions and aimed to evaluate whether the features could differentiate between GPA, SBO, and NPC involving the skull base. We retrospectively evaluated the MRI findings of patients with GPA, SBO, and NPC. We performed univariable logistic regression analyses to identify the predictive variables for differentiating between conditions and evaluated their diagnostic values. We showed, for the first time, that certain MRI findings significantly differed between patients with GPA and those with SBO or NPC, including the lesion morphology and extent, the apparent diffusion coefficient (ADC) values, the contrast enhancement patterns, the presence or absence of necrosis, and retropharyngeal lymphadenopathy. In conclusion, utilizing certain MRI features can improve the diagnostic performance of MRI by differentiating GPA with skull base involvement from other conditions with similar radiologic findings, including SBO and NPC, facilitating treatment plans and, thus, improving patient outcomes.

## 1. Introduction

Granulomatosis with polyangiitis (GPA), previously known as Wegener’s granulomatosis, is one of the most frequently encountered types of otolaryngological vasculitis [1,2]. GPA is a multisystemic antineutrophil cytoplasmic antibody (ANCA)-associated vasculitis characterized by the presence of necrotizing granulomatous inflammation that affects small- or medium-sized vessels [2,3]. Its diagnosis is typically based on clinical manifestations and positive serum ANCA tests [1,3,4,5,6].

Approximately 72–99% of patients with systemic GPA present with head and neck manifestations [1,3,4]. In particular, primary otologic symptoms, such as tympanomastoid effusion, are observed in up to 40% of patients with GPA, and conducive or sensorineural hearing loss affects 19–61% of patients [1,7,8]. Otologic impairment in patients with GPA can develop when the disease involves the skull base or the Eustachian tubes, and it is often accompanied by nasopharyngeal manifestations [8]. Therefore, in such cases, it can be challenging to discriminate GPA from the infectious processes driving skull base osteomyelitis (SBO) and the malignant processes involving the nasopharynx [9,10,11,12,13,14,15,16,17,18]. Although serum ANCA screening can provide evidence for the diagnosis of GPA, this test is not routinely performed, but selectively ordered for the patients with clinical suspicion in the daily practice. Moreover, the reported sensitivities of ANCA testing for diagnosing GPA range from 34 to 92% (pooled sensitivity, 66%). Therefore, ANCA testing alone cannot always distinguish between GPA and the other two conditions [14,19].

Several case reports have described magnetic resonance imaging (MRI) findings in patients with these three disease categories; however, their imaging characteristics mimic each other and the results have been shown to be inconclusive [9,10,11,12,13,14,15,16,17,18]. To our knowledge, few studies have investigated the discriminative value of MRI in differentiating GPA from infective osteomyelitis and nasopharyngeal carcinoma (NPC) involving the skull base. Despite the overlap, we hypothesized that certain MRI characteristics might differ significantly according to the diagnosis. Therefore, the purpose of this study was to evaluate whether MRI can be used to differentiate between GPA, SBO, and NPC involving the skull base.

## 2. Materials and Methods

### 2.1. Ethics

This study was conducted in accordance with the Declaration of Helsinki. The Institutional Review Board of our hospital approved this retrospective study (B-2107-696-108, approval date 9 July 2021). The requirement for informed consent was waived by the board because of the retrospective nature of the study.

### 2.2. Study Population

We searched the database of our tertiary referral institution for patients who received a confirmative diagnosis of GPA based on clinical examinations, serological testing for ANCA, and/or histopathological assessments conducted between January 2004 and April 2021. Among the identified patients, those who manifested otologic symptoms and, thus, underwent high-resolution MRI that included the skull base and temporal bone, using a 3T scanner, were selected for the inclusion. A board-certified neuroradiologist (Y.J.B., with 11 years of experience in head and neck radiology) reviewed all the MRI data and identified patients with disease involvement in the skull base and/or Eustachian tube. As a result, patients with GPA involving the skull base who underwent high-resolution MRI were finally included in the GPA group.

During the same period, patients who were diagnosed with spontaneous SBO were identified from the institutional database. Among such patients, those who underwent high-resolution skull base and temporal bone 3T MRI, as well as histopathological evaluations of the skull base lesion that could exclude the diagnosis of GPA, were included in the SBO group. Lastly, we identified patients who were diagnosed with NPC involving the skull base and included them in the NPC group. To achieve this goal, we selected patients with histopathologically confirmed NPC who underwent high-resolution MRI that included the skull base and temporal bone prior to initiating treatment, and whose clinical tumor stage was at least T3, according to the 8th edition of the American Joint Committee on Cancer (AJCC) staging system, to ensure the skull base involvement of the tumor [20]. With regard to all three groups, patients who did not undergo high-resolution MRI on a 3T scanner, those whose MRI did not cover the skull base and temporal bone, those who had motion or metallic artifacts on the MRI, and those who presented with skull base pathologies other than GPA, SBO, and NPC were excluded.

### 2.3. MRI Protocol

MRI was performed using a 3T scanner (Achieva and Ingenia, Philips Healthcare, Best, The Netherlands) with a 16- or 32-channel SENSE head coil (Philips Healthcare). For evaluating the skull base, the following sequences and imaging parameters were utilized: (a) Coronal T2-weighted imaging (T2WI) with fat suppression (repetition time [TR], 2500 ms; echo time [TE], 80 ms; field-of-view (FOV), 200 × 200 mm^2^; acquisition matrix, 400 × 400; slice thickness, 4 mm); (b) Axial T2WI with and without fat suppression (TR, 3300 ms; TE, 80 ms; FOV, 180 × 220 mm^2^; acquisition matrix, 440 × 440; slice thickness, 3 mm); and (c) Axial T1-weighted imaging (T1WI) without fat suppression (TR, 690 ms; TE, 15 ms; FOV, 180 × 220 mm^2^; acquisition matrix, 440 × 440; slice thickness, 3 mm). Following the intravenous administration of a bolus of gadobutrol, a gadolinium-based contrast agent (Gadovist^®^, 0.1 mmol/kg; Bayer Healthcare, Berlin, Germany), contrast-enhanced T1WI with fat suppression in the axial (TR, 600 ms; TE, 15 ms; FOV, 180 × 220 mm^2^; acquisition matrix, 440 × 440; slice thickness, 3 mm) and coronal (TR, 550 ms; TE, 15 ms; FOV, 200 × 200 mm^2^; acquisition matrix, 400 × 400; slice thickness, 4 mm) planes was obtained. For patients with an initial clinical suspicion of malignancy, additional diffusion-weighted imaging (DWI) was performed using the following parameters: b-values of 0 and 1000 s/mm^2^; three orthogonal diffusion gradients: TR, 6400 ms; TE, 65 ms; FOV, 220 × 220 mm^2^; acquisition matrix, 128 × 128; and slice thickness, 3 mm.

### 2.4. MRI Analysis

Two neuroradiologists (Y.J.B. with 11 years of experience and B.S.C. with 21 years of experience), who were blinded to the clinical information and pathological results of each patient, independently reviewed all of the MRI scans. After the independent evaluations, both radiologists reviewed the MRI scans by consensus and any discrepancies were resolved by a third neuroradiologist (S.J.C. with 10 years of experience).

The imaging findings were evaluated based on the following features: (a) Morphology (lesion location, shape, and margin); (b) Inner nature (signal intensity and necrosis); (c) Extent (invasion of adjacent structures, perineural involvement, and intracranial extension); and (d) Associated retropharyngeal lymphadenopathy. The location was determined by the epicenter of the soft-tissue mass-forming area and was classified as the diploic space of the skull base, nasopharyngeal wall, parapharyngeal space, or lateral structures including the tympanomastoid bone and parotid gland. The shape was defined as round/oval, lobular, or irregular. The margin was classified as well-defined (>2/3 of the border being sharply demarcated), partially defined (between 1/3 and 2/3 of the border), and poorly defined (<1/3 of the border). The signal intensity of the lesion on T1WI and T2WI was characterized in comparison with that of the adjacent muscle as hypointense, isointense, or hyperintense. If DWI data were available, the quantitative value of the apparent diffusion coefficient (ADC, ×10^−3^ mm^2^/s) was measured. To measure the ADC value, two neuroradiologists (Y.J.B. and B.S.C.) allocated regions-of-interest in the enhancing lesion while avoiding the necrotic portion. The values were then averaged for further statistical analysis. The patterns of the contrast enhancement of the lesion were classified as homogeneous, heterogeneous, or internally necrotic, based on the hypointense T1 and hyperintense T2 signal intensity. For the quantitative assessment of the contrast enhancement, a neuroradiologist (Y.J.B.) measured the signal intensity of the enhancing portion of the lesion on contrast-enhanced T1WI by allocating the smoothed polygonal regions-of-interest, attempting to include as much of the lesion as possible while excluding the nonenhancing necrotic portion. Then, the signal intensity of the pons was measured in the same manner for the normalization. Since the pons has a relatively large yet homogenous area of the structure, it can provide a reliable measurement of the signal intensity among the infratentorial structures [21,22]. Lastly, the enhancing ratio of the lesion was defined as the signal intensity of the enhancing portion divided by that of the pons. Next, the invasion of the following adjacent structures was evaluated using contrast-enhanced T1WI with reference to T2WI: the Eustachian tube (outside of the nasopharynx); the external auditory canal; the prevertebral muscle the infratemporal fossa; the retroclival area; the cavernous sinus; the dura; and the cranial nerve. Finally, the associated lymphadenopathy in the retropharyngeal station was assessed, with abnormal retropharyngeal lymph node enlargement defined as a lymph node with a short diameter, exceeding 0.5 cm [23].

### 2.5. Statistical Analysis

The clinical findings were compared between the GPA, SBO, and NPC groups using nonparametric tests. For the MRI findings, the interobserver agreement between the two radiologists was calculated based on the interclass correlation coefficient (ICC) and Cohen’s kappa (κ) index. The agreement was considered to be excellent when the ICC and κ values exceeded 0.75 and 0.8, respectively. Using consensus reading, the MRI findings were compared between the GPA, SBO, and NPC groups using the chi-squared test or Fisher’s exact test for the categorical variables, and the Kruskal–Wallis test for the continuous variables. In particular, the MRI findings were compared between the GPA and SBO groups, and between the GPA and NPC groups, to investigate the ability of MRI to discriminate GPA from the other two pathologies using the chi-squared test or the Fisher’s exact test, and the Mann–Whitney U test. Next, we utilized univariable and/or multivariable logistic regression with Firth correction [24] analysis to identify specific MRI findings that could be used to differentiate GPA from SBO and NPC (each and combined). Finally, the diagnostic performance (i.e., the sensitivity, specificity, positive predictive value, and negative predictive value) was quantified. Statistical significance was set at *p* < 0.05. Statistical analyses were performed using Statistical Package for the Social Sciences (SPSS) (version 17.0; SPSS, Chicago, IL, USA), MedCalc 17.9, (MedCalc, Mariakerke, Belgium), and SAS (version 9.3; SAS Institute, Cary, NC, USA) software.

## 3. Results

### 3.1. Clinical Characteristics

A total of 55 patients were included in the study. A total of 15 patients were included in the GPA group (six men and nine women; median age, 63 years; age range, 38–91 years). All patients in the GPA group had positive ANCA serological test results, and histopathological confirmation was performed in 12 patients via nasopharyngeal biopsy (*n* = 4), paranasal sinus biopsy (*n* = 2), lung biopsy (*n* = 2), external auditory canal biopsy (*n* = 1), meninx biopsy (*n* = 1), kidney biopsy (*n* = 1), or eyelid biopsy (*n* = 1). The SBO group was comprised of 24 patients (twenty men and four women; median age, 73 years; age range, 38–86 years). The NPC group consisted of 16 patients (twelve men and four women; median age, 50 years; age range, 37–80 years) who were at clinical stages T3 (*n* = 11) or T4 (*n* = 5). Age and sex significantly differed among the three groups (*p* = 0.016 and < 0.001, respectively). In particular, the NPC group was significantly younger than the GPA and SBO groups (*p* = 0.019 and < 0.001, respectively). Although there were higher proportions of men in the SBO and NPC groups than in the GPA group, there were no statistically significant differences between any two groups with respect to sex (*p* > 0.05).

### 3.2. Interobserver Agreement on MRI Findings

The interobserver agreements were excellent for the one continuous variable of the ADC value (ICC 0.91; confidence interval, 0.81–0.96), and for all the categorical variables (κ > 0.8 for all variables).

### 3.3. Comparison of MRI Characteristics between the GPA, SBO, and NPC Groups

The MRI findings for each group are detailed in Table 1.

#### 3.3.1. Morphology

The epicenter of the lesion was mostly within the parapharyngeal space in the GPA group, whereas in the SBO group, the lesions were mostly centered in the diploic space of the skull base, and in the NPC group, the lesions were mostly centered within the nasopharyngeal wall. In the GPA and SBO groups, the shapes of the lesions were mostly irregular, whereas, in the NPC group, the shape was predominantly lobular. The lesion margins were mostly poorly defined in the GPA and SBO groups, while the NPC group showed lesions that were mostly partially defined. Overall, the locations, shapes, and margins of the lesions significantly differed between the GPA and NPC groups, whereas no significant differences were observed between the GPA and SBO groups.

#### 3.3.2. Inner Nature

**Figure 1:**
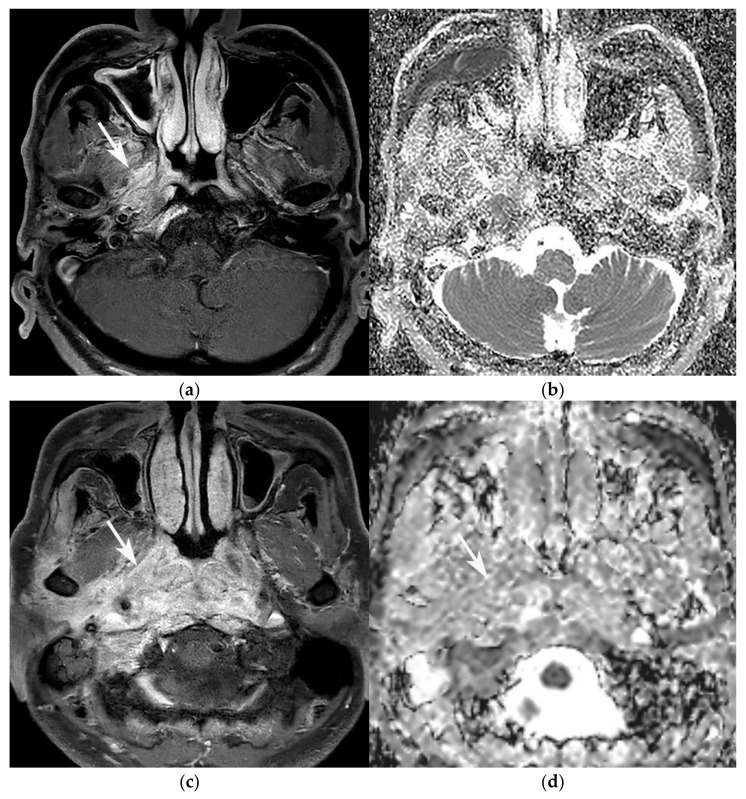
Comparison of ADC values between GPA lesion (**a**,**b**), and SBO lesion (**c**,**d**). (**a**) Axial contrast-enhanced T1WI of a 77-year-old male diagnosed with GPA shows infiltrative enhancing lesion (arrow) involving the right-side nasopharynx, parapharyngeal space, prevertebral muscle, and skull base. (**b**) His ADC map shows a relatively low ADC value inside the enhancing lesion (arrow). The measured value was 0.71 × 10^−3^ mm^2^/s. (**c**) On axial contrast-enhanced T1WI, a 79-year-old male diagnosed with SBO shows large area of infiltrative enhancing lesion (arrow) involving the nasopharynx, parapharyngeal space, carotid space, prevertebral and retropharyngeal space, skull base, and adjacent infratemporal fossa. (**d**) On his ADC map, the lesion shows relatively high ADC values inside the enhancing lesion (arrow), which was measured as 1.56 × 10^−3^ mm^2^/s.

Most of the lesions exhibited hypointense T1 signal intensity, and hyperintense T2 signal intensity, in all three groups, and no significant differences were observed. Among the 28 patients who had available DWI and ADC maps, the ADC of the lesions in the SBO group was significantly higher than that of the lesions in the GPA and NPC groups. The pattern of contrast enhancement was mostly heterogeneous in the SBO group, whereas it was predominantly homogeneous in the GPA and NPC groups. However, there was no significant difference in the enhancing ratio values between GPA, SBO, and NPC. Finally, internal necrosis was not observed in most patients with GPA or NPC, whereas it was present in many patients with SBO. Therefore, the ADC values, the patterns of contrast enhancement, and the presence of internal necrosis all significantly differed between the GPA and SBO groups (Figure 1), but not between the GPA and NPC groups.

#### 3.3.3. Extent

**Figure 2:**
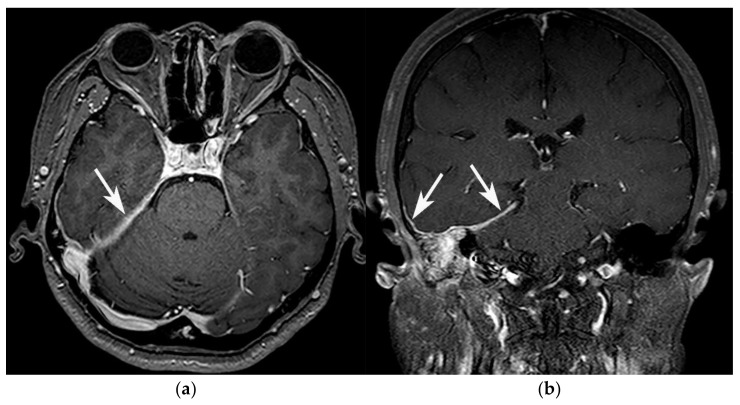
Dural involvement in a 54-year-old female patient diagnosed with GPA. (**a**) Axial, and (**b**) coronal contrast-enhanced T1WI show enhancing dural thickening at right temporal convexity and right tentorium (arrows).

Most cases of GPA and SBO involve the Eustachian tube, the external auditory canal, and the dura, whereas the involvement of these regions was observed in a smaller portion of the NPC cases (Figure 2). Statistically, these findings significantly differed between the GPA and NPC groups, but not between the GPA and SBO groups. All three groups exhibited a high incidence of prevertebral muscle involvement, and no significant differences were observed between the groups. None of the patients with GPA, and only one with NPC, exhibited infratemporal fossa involvement, whereas it was observed in nine SBO cases; therefore, the involvement of the infratemporal fossa significantly differed between the GPA and SBO groups, but not between the GPA and NPC groups. The incidences of retroclival and cavernous sinus involvement did not differ significantly between the three groups. Cranial nerve involvement was observed in nearly 50% of the patients with GPA. Although it was observed in a much lower proportion of patients with NPC or SBO (31% and 25%, respectively), and the differences were not statistically significant.

#### 3.3.4. Associated Lymphadenopathy

**Figure 3:**
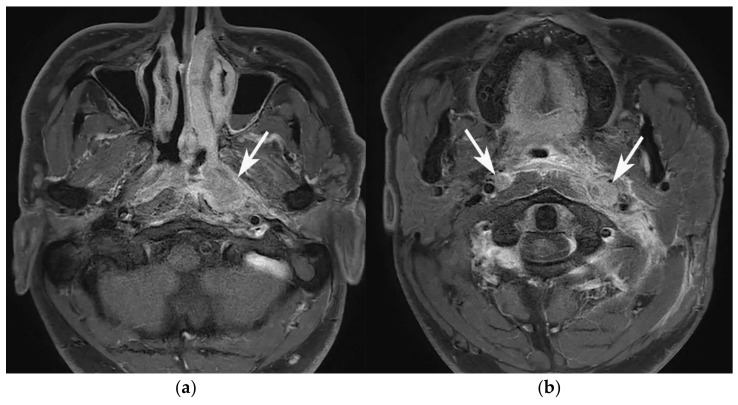
Retropharyngeal lymphadenopathy in a 50-year-old male patient diagnosed with NPC. (**a**) Axial contrast-enhanced T1WI shows enhancing mass-forming lesion in the left posterolateral nasopharynx (arrow). (**b**) In this patient, bilateral retropharyngeal lymph nodes are enlarged (arrows), suggesting retropharyngeal nodal metastases.

Retropharyngeal lymphadenopathy occurred significantly more frequently in patients with NPC than in those with GPA or SBO (Figure 3). There was no significant difference between the GPA and SBO groups.

### 3.4. Diagnostic Performance of MRI Characteristics

All of the variables of the MRI findings were utilized in the logistic regression analysis, except for certain variables for the following reasons: (a) Since the GPA lesions were mostly centered in the parapharyngeal space, we dichotomized four subsites of the location (i.e., the diploic space of the skull base, the nasopharyngeal wall, the parapharyngeal space, and the lateral structures) into the parapharyngeal space and the nonparapharyngeal space; (b) Since there was overlap in the shapes and the margins of the lesions of the MRI findings, we chose to compare the margins rather than the shapes; and (c) Since there was overlap between the locations and extent of the lesions, in terms of the involvement of the Eustachian tube and the external auditory canal (i.e., the lesions that involved the Eustachian tube were mostly centered in the parapharyngeal space, and those that involved the external auditory canal were mostly centered at the lateral structures), we chose to compare the locations rather than the involvement of the Eustachian tube and the external auditory canal.

#### 3.4.1. Differentiation between GPA and SBO

**Figure 4:**
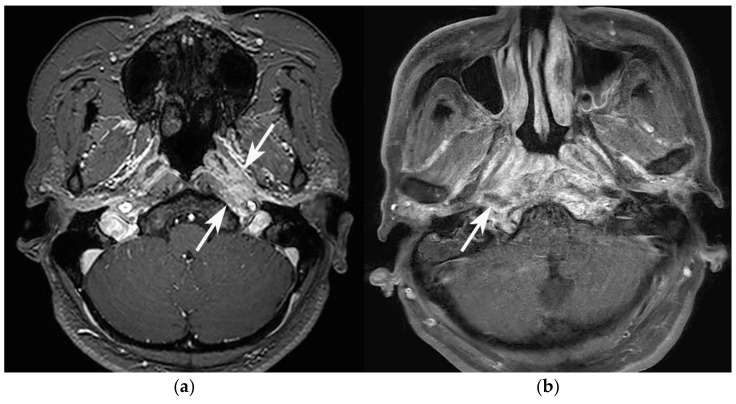
Comparison between GPA and SBO. (**a**) On axial contrast-enhanced T1WI of a 45-year-old female, GPA lesion shows relatively homogeneously enhancing infiltrative lesion (arrows) centered in the left side parapharyngeal space, skewed laterally from the nasopharynx. (**b**) SBO lesion of a 60-year-old male shows heterogeneously enhancing infiltrative lesion involving bilateral nasopharynx, prevertebral space, and skull base. Notably, focal internal necrosis (arrow) is seen in the right skull base area.

A parapharyngeal epicenter, homogeneous contrast enhancement, and the absence of internal necrosis were significant predictors for the differential diagnosis of GPA over SBO (Figure 4, Table 2). The diagnostic performances of these three variables are summarized in Table 3. Homogeneous contrast enhancement provided relatively high specificity (95.8%) and positive predictive value (88.9%), and the absence of internal necrosis provided relatively high sensitivity (86.7%) and negative predictive value (86.7%).

#### 3.4.2. Differentiation between GPA and NPC

**Figure 5:**
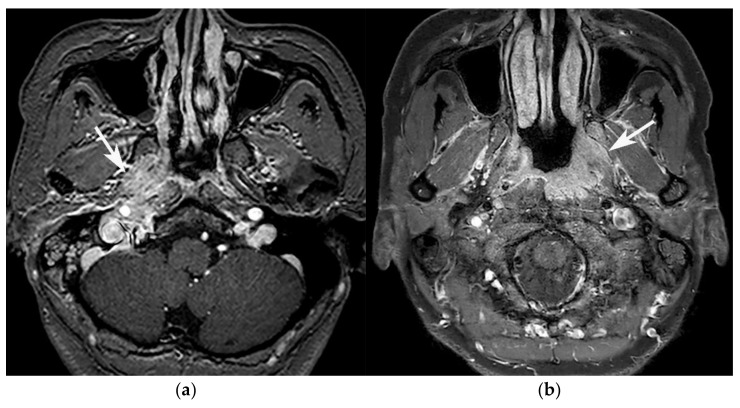
Comparison between GPA and NPC. (**a**) Axial contrast-enhanced T1WI of a 54-year-old female shows GPA lesion centered in the right parapharyngeal space, skewed laterally from the nasopharynx (arrow). Its infiltrative enhancement causes a poorly defined margin. (**b**) On the contrary, NPC lesion in a 46-year-old female shows enhancing lesion in the right lateral and posterior nasopharynx (arrow). Although it extends into adjacent parapharyngeal space, the lesion is centered in the nasopharyngeal wall. In addition, the margin of NPC is relatively well-defined compared to that of GPA lesion.

A lesion epicenter in the parapharyngeal space, a poorly defined margin, dural involvement, and the absence of retropharyngeal lymphadenopathy were significant predictors for the differential diagnosis of GPA over NPC (Figure 5, Table 4). The diagnostic performances of these four variables are summarized in Table 5. A parapharyngeal epicenter, a poorly defined margin, and dural involvement provided high diagnostic performance; in particular, dural involvement was the variable with the highest sensitivity (73.3%), specificity (93.8%), positive predictive value (91.7%), and negative predictive value (78.9%).

#### 3.4.3. Differentiation between GPA and Non-GPA (SBO and NPC Combined)

For the univariable analysis, a lesion epicenter in the parapharyngeal space and dural involvement were the significant predictors for differentiating GPA from SBO and NPC combined (Table 6). When performing subsequent multivariable analyses, the parapharyngeal epicenter was the only significant predictor for this differentiation, with a sensitivity of 66.7%, a specificity of 77.5%, a positive predictive value of 52.6%, and a negative predictive value of 86.1% (Table 6 and Table 7).

## 4. Discussion

Our study is the first to identify certain MRI findings that could provide diagnostic value for differentiating GPA from SBO or NPC. The findings that could differentiate between GPA and SBO were a lesion with a parapharyngeal epicenter, homogeneous contrast enhancement, and the absence of necrosis. For differentiating between GPA and NPC, the important MRI findings were a lesion with a parapharyngeal epicenter, a poorly defined margin, dural involvement, and the absence of retropharyngeal lymphadenopathy.

It is often challenging to diagnose GPA in clinical practice when patients present with nonspecific symptoms [9,10,11,12,13,14,15,16,17,18]. In particular, when GPA affects the head and neck, the absence of symptom specificity and the clinical variability of the head and neck manifestations often contribute to the misdiagnosis of the disease as an infectious or neoplastic condition [3,9,10,11,12,13,14,15,16,17,18,25]. Patients with GPA involving the skull base and/or the Eustachian tube may manifest otologic symptoms mainly, but possibly accompanied by constitutional symptoms and a high serologic inflammatory marker, such as the erythrocyte sedimentation rate (ESR) [15,17], which may clinically mimic SBO. Similarly, both skull-base-involving GPA and NPC can share clinical presentations, such as middle ear and mastoid effusion, hearing loss, otalgia, and/or cranial nerve involvement [9,10,11,12,13]. Particularly, the otologic manifestation of conductive hearing loss can occur in patients with GPA because of nasopharyngeal inflammation, and sensorineural hearing loss can be attributed to cranial nerve impairment [3,26,27]. A delayed diagnosis of GPA in such cases can be disastrous since GPA with concomitant neural or temporal disorders requires early and aggressive therapy [28]. Correctly diagnosed early-stage GPA can be effectively treated with corticosteroid, methotrexate, and/or cyclophosphamide therapy [3]. Therefore, the early and accurate diagnosis of head and neck GPA can improve the treatment outcomes of patients.

Unfortunately, radiological findings may lead to an erroneous diagnosis as well because the radiologic features of GPA often overlap with those of other diseases [9,10,11,12,13,14,15,16,17,18,29], delaying the time to diagnosis [3,23,29,30,31]. Accordingly, it is not surprising that there have been several case reports published on GPA cases in which the MRI findings mimicked those of SBO or NPC [9,10,11,12,13,14,15,16,17,18,29], or that, to date, no studies have identified MRI findings possessing diagnostic value for differentiating between these diseases. Nevertheless, the advent of high-resolution MRI has facilitated a more detailed assessment of the imaging characteristics and the extent of such lesions, and the diagnostic role of MRI is expanding. On the basis of these factors, we hypothesized that there could be specific MRI findings that could be utilized for differential diagnoses.

Thus, in this study, we systemically reviewed high-resolution 3T MRI scans from patients with GPA, SBO, and NPC involving the skull base. We focused on identifying the MRI features that could allow for the differentiation between GPA and non-GPA (SBO and NPC, each and combined). Ultimately, we identified several MRI characteristics that could assist in predicting GPA over the other two pathologies. First, the epicenters of the lesions were distinct for each of the three pathologies; in GPA, the lesion was predominantly localized within the parapharyngeal space, whereas, in SBO and NPC, the lesions were predominantly localized within the skull base diploic space and the nasopharyngeal wall, respectively. This result seems obvious considering the nature of the pathologies. Notably, when GPA caused otologic symptoms and involved the skull base, the enhancing soft-tissue mass-like lesions were mostly located in the parapharyngeal space; thus, their presence could involve the Eustachian tube, but they did not exist within the nasopharynx or bone structures per se. This skewed epicenter from nasopharynx was a significant predictor for GPA over NPC and was a variable that could provide high diagnostic performance. When we retrospectively applied this finding to the previous case reports of GPA mimicking NPC [9,10,11,12,13], we found that the previous GPA cases also involved the parapharyngeal space. Furthermore, when we assessed the predictive MRI features that could differentiate GPA from non-GPA (SBO and NPC combined), the parapharyngeal epicenter proved to be the only significant predictor. Therefore, we believe that a simple assessment of a lesion’s epicenter could improve the radiologic diagnosis of GPA using MRI. Second, the margin of the lesion was mostly partially defined in cases of NPC, whereas it was mostly poorly defined in cases of GPA and SBO. Since GPA and SBO are characterized by inflammatory pathologies, they must be considered infiltrative in nature [8,14,15,16,17,18,29]. In contrast, NPC lesions are typically more defined; however, since we enrolled patients with NPC of clinical tumor stage 3 or higher, most of the NPC lesions were only partially defined, probably due to the skull base infiltration. Accordingly, the poor margin led to the inability to differentiate between GPA and SBO, but it was possible to distinguish GPA from NPC with high diagnostic performance. Third, as for the T1 and T2 signal intensities, most of the three pathologies showed hypointense T1 and hyperintense T2 signal intensities, which could not assist in a differential diagnosis. Nonetheless, the ADC values significantly differed between the GPA and SBO groups. It is well-known that ADC values can reflect the degree of water diffusion inside the tissue, and low ADC values are indicative of restricted diffusion resulting from a high cellularity [29]. Our results revealed higher ADC values in the SBO group than in the NPC group, which is in accordance with the findings of previous studies [32,33,34]. Inflammatory processes may result in higher diffusion than malignant neoplastic processes [29]. Nonetheless, this finding did not reveal itself to be a significant predictor in the univariable logistic regression analysis. Interestingly, in the present study, the GPA group exhibited a low ADC value that was comparable to that of the NPC group. Although GPA is an inflammatory disease involving vasculitis, necrotizing granulomas are a known component of its pathology, along with abundant liquefactive or coagulative necrosis, all of which can demonstrate a low ADC value [35]. Therefore, the ADC value could not be used to differentiate GPA from NPC. Fourth, the pattern of contrast enhancement was mostly heterogeneous in SBO and mostly homogeneous in NPC, whereas 53.3% and 46.7% of patients with GPA exhibited homogeneous or heterogeneous enhancement, respectively. This can be understood within the same context as the fact that grossly visible necrosis was present in 54.2% of SBO cases, while necrosis was not present in most of the GPA and NPC cases. Infectious SBO can result in abscesses, a feature that can support its diagnosis [29], which can explain the more frequent heterogeneous enhancement in patients with SBO, along with the more frequent findings of necrosis. Although GPA may also result in necrosis, it is often accompanied by microscopic necrosis [35]. Accordingly, many of the GPA cases exhibited heterogeneous enhancement, but gross necrosis was less commonly visible. As a result, homogeneous contrast enhancement and the absence of necrosis were significant predictors of GPA over SBO. Although these two variables significantly differed between the GPA and NPC groups as well, they were not significant factors in the univariable logistic regression analysis. Fifth, regarding the lesion extent assessed by the contrast-enhancing lesions, infratemporal fossa involvement was observed more frequently in patients with SBO than in those with GPA, and Eustachian tube/external auditory canal/dura involvement was more common in patients with GPA than in those with NPC. In particular, the univariable logistic regression analysis revealed that dural involvement was a strong predictor of GPA over NPC, and the diagnostic performance of the variable was high. Meningeal inflammation is a rare but well-known manifestation of GPA [36]. The pathogenesis of pachymeningitis in GPA is also driven by granulomatous inflammation, and it is more frequently observed in the early disease course of clinically active limited GPA [35,36]. In addition, in most cases of meningeal disease, cranial nerve involvement has been reported; this includes the 2nd, 3rd, 6th, or 7th nerves, and the involvement of the 8th nerve can result in hearing loss [13,16,35,36]. Considering that the involvement of the dura typically occurs in a far-advanced stage of NPC, there is no doubt that dural involvement is a significant predictor of GPA over NPC. Meanwhile, many of the SBO cases also exhibited dural thickening and enhancement adjacent to the main inflammatory lesion by direct extension. The mechanism for this dural involvement in SBO differs from that of GPA, which is a remote granulomatous inflammation. Nonetheless, the presence of dural thickening and enhancement itself cannot be used to differentiate between GPA and SBO. Lastly, the prevalence of retropharyngeal lymphadenopathy was significantly higher in patients with NPC. NPC is known to accompany retropharyngeal nodal metastasis starting from the early stage of the tumor [37]; thus, retropharyngeal lymphadenopathy (i.e., metastasis) is a frequent occurrence. In contrast, the manifestation of cervical lymphadenopathy in GPA is possible, but rare [38]. In addition, lymph node enlargement can occur in SBO as a reactive change induced by inflammation, but retropharyngeal lymph node involvement was not frequent in our study. As a result, the presence of retropharyngeal lymphadenopathy could differ between GPA and NPC, and this variable had a high sensitivity, although its specificity was low, and it could not differentiate between GPA and SBO.

Clinically, it is important to be aware that SBO can be superimposed upon GPA [39]. Von Itzstein et al. [39] have reported several cases of GPA superinfected by SBO, and emphasized that, although diagnostically challenging, SBO should be excluded before initiating immunosuppression for GPA. In this regard, our study results can assist in the discrimination of SBO, regardless of underlying GPA, and, thus, can help in making appropriate treatment plans. Additionally, even when MRI findings indicate SBO, any remaining lesions on the MRI after treating SBO should be carefully evaluated for the possibility of underlying GPA. Therefore, we believe that follow-up MRI examinations and close monitoring, incorporating clinical examination and the laboratory test, should be conducted so as not to overlook GPA.

Our study had several limitations. First, the number of participants was relatively small and may possess selection bias. Considering that we identified feasible patients from our nationwide tertiary referral hospital over a period of more than a decade, the rarity of head and neck GPA and the low disease prevalence might be fundamental limiting factors. Further studies with a larger number of subjects included from the multicenter are necessary. Second, we did not consider EBV-DNA testing for the diagnosis of NPC before the radiologic evaluation in our study. Although EBV-DNA testing in the nasopharyngeal tissue or plasma may help in diagnosing NPC [40], the presence of EBV-DNA in the nasopharynx cannot confirm the diagnosis of NPC, since EBV-DNA can be present in the normal nasopharynx, particularly in the endemic areas, such as Far East Asia [41], and also since it can be detected in other cancer types, such as Hodgkin lymphoma [42]. Rather, we believe that the radiologic evaluation should precede in order to provide accurate differential diagnosis, and that further diagnostic processes, such as nasopharyngeal biopsy, should be followed for NPC. Finally, the DWI data were lacking in some patients, which could have limited the assessment of the diagnostic value of DWI in differentiating GPA from SBO and NPC. Therefore, high-resolution MRI using advanced imaging techniques should be adopted in the future.

## 5. Conclusions

Certain high-resolution 3T MRI findings significantly differed between patients with GPA and those with SBO or NPC, and those features could be useful for differential diagnoses. These characteristics included the lesion morphology, such as the location and margin, the ADC value, the pattern of contrast enhancement, the presence or absence of necrosis, the extent of the lesion, and the presence or absence of retropharyngeal lymphadenopathy. More specifically, a parapharyngeal epicenter, homogeneous contrast enhancement, and the absence of necrosis were significant discriminators between GPA and SBO, whereas a parapharyngeal epicenter, a poorly defined margin, dural involvement, and the absence of retropharyngeal lymphadenopathy were significant discriminators for GPA over NPC. In conclusion, utilizing certain MRI features can improve the diagnostic performance of MRI in differentiating GPA involving the skull base from other conditions that mimic each other, including SBO and NPC involving the skull base.

## Figures and Tables

**Table 1:** Radiologic findings of skull base pathologies based on high-resolution MRI.

Findings	GPA (*n* = 15)	SBO (*n* = 24)	NPC (*n* = 16)	*p* Value ^1^	*p* Value ^2^	*p* Value ^3^
Morphology						
Location				0.101	0.16	0.019 *
Skull base diploic space	2 (13.3%)	12 (50.0%)	0 (0.0%)			
Nasopharyngeal wall	1 (6.7%)	0 (0.0%)	13 (81.3%)			
Parapharyngeal space	10 (66.7%)	6 (25.0%)	3 (18.8%)			
Lateral structures ^4^	2 (13.3%)	6 (25.0%)	0 (0.0%)			
Shape				0.002 *	0.279	0.007 *
Round/oval	0 (0.0%)	0 (0.0%)	1 (6.3%)			
Lobular	3 (20.0%)	1 (4.2%)	10 (25.5%)			
Irregular	12 (80.0%)	23 (95.8%)	5 (31.3%)			
Margin				<0.0001 *	0.062	0.002 *
Well-defined	0 (0.0%)	0 (0.0%)	3 (18.8%)			
Partially defined	4 (26.7%)	1 (4.2%)	10 (62.5%)			
Poorly defined	11 (73.3%)	23 (95.8%)	3 (18.8%)			
**Inner nature**						
T1 signal intensity				0.053	0.142	0.137
Hypointense	13 (86.7%)	24 (100.0%)	16 (100.0%)			
Isointense	2 (13.3%)	0 (0.0%)	0 (0.0%)			
Hyperintense	0 (0.0%)	0 (0.0%)	0 (0.0%)			
T2 signal intensity				0.28	0.142	0.654
Hypointense	0 (0.0%)	0 (0.0%)	0 (0.0%)			
Isointense	2 (13.3%)	0 (0%)	4 (25.0%)			
Hyperintense	13 (86.7%)	24 (100%)	16 (100.0%)			
ADC (×10^−3^ mm^2^/s)	0.84 (0.71-0.94)	1.18 (0.79–2.37)	0.76 (0.61–1.77)	0.001 *	0.001 *	0.428
Contrast enhancement				<0.0001 *	0.001 *	0.473
Homogeneous	8 (53.3%)	1 (4.2%)	11 (68.8%)			
Heterogeneous	7 (46.7%)	23 (95.8%)	5 (31.3%)			
Enhancing ratio	1.94 (1.43–2.98)	1.95 (1.25–3.04)	1.83 (1.48–2.54)	0.665	0.875	0.446
Necrosis				0.005 *	0.017 *	1
Absent	13 (86.7%)	11 (45.8%)	14 (87.5%)			
Present	2 (13.3%)	13 (54.2%)	2 (12.5%)			
**Extent ^5^**						
Eustachian tube	13 (86.7%)	21 (87.5%)	3 (18.8%)	<0.0001 *	1	<0.0001 *
External auditory canal	8 (53.3%)	14 (58.3%)	1 (6.3%)	0.003 *	1	0.006 *
Prevertebral muscle	11 (73.3%)	19 (79.2%)	15 (93.8%)	0.306	0.711	0.172
Infratemporal fossa	0 (0.0%)	9 (37.5%)	1 (6.3%)	0.706	0.008 *	0.333
Retroclival area	3 (20.0%)	10 (41.7%)	0 (0.0%)	0.175	0.295	0.064
Cavernous sinus	3 (20.0%)	5 (20.8%)	1 (6.3%)	0.429	1	0.333
Dura	11 (73.3%)	16 (66.7%)	1 (6.3%)	<0.0001 *	0.734	<0.0001 *
Cranial nerve	7 (46.7%)	6 (25.0%)	5 (31.3%)	0.37	0.185	0.473
**Retropharyngeal lymphadenopathy**	3 (20.0%)	1 (4.2%)	13 (81.3%)	<0.0001 *	0.279	0.001 *

Continuous variables are expressed as the median (range). ^1^ *p* values from the comparison between three groups. ^2^ *p* values from the comparison between the GPA and SBO groups. ^3^ *p* values from the comparison between the GPA and NPC groups. ^4^ Including the tympanomastoid bone and parotid gland. ^5^ Numbers (%) involving the following structures. * *p* values less than 0.05 indicate statistical significance. MRI, magnetic resonance imaging; GPA, granulomatous polyangiitis; SBO, skull base osteomyelitis; NPC, nasopharyngeal carcinoma; ADC, apparent diffusion coefficient.

**Table 2:** Univariable analysis for differentiating GPA from SBO.

Variables	OR	95% CI	*p* Value
Location			
Parapharyngeal *over* nonparapharyngeal space	5.43	1.34–22.05	0.018 *
Margin			
Partially *over* poorly defined	6.13	0.72–52.46	0.098
T1 signal intensity			
Isointense *over* hypointense	9.08	0.21–397.72	0.253
T2 signal intensity			
Isointense *over* hyperintense			
ADC (×10^−3^ mm^2^/s)	<0.001	<0.001–6.03	0.092
Contrast enhancement			
Heterogeneous *over* homogeneous	0.056	0.008–0.42	0.005 *
Necrosis			
Present *over* absent	0.16	0.032–0.79	0.024 *
Extent			
Prevertebral muscle	0.72	0.16–3.26	0.67
Infratemporal fossa	0.053	0.002–1.15	0.061
Retroclival area	0.39	0.089–1.69	0.206
Cavernous sinus	0.99	0.20–4.89	0.993
Dura	1.32	0.32–5.42	0.703
Cranial nerve	2.51	0.64–0.87	0.187
Retropharyngeal lymphadenopathy	4.39	0.46–41.55	0.198

* *p* values less than 0.05 indicate statistical significance. OR, odds ratio; CI, confidence interval; GPA, granulomatous polyangiitis; SBO, skull base osteomyelitis; ADC, apparent diffusion coefficient.

**Table 3:** Diagnostic performances of variables for differentiating GPA from SBO.

Variables	Sensitivity	Specificity	Positive Predictive Value	Negative Predictive Value
Parapharyngeal epicenter	66.7%	25.0%	35.7%	54.5%
Homogeneous enhancement	53.3%	95.8%	88.9%	76.7%
Absence of necrosis	86.7%	54.2%	54.2%	86.7%

GPA, granulomatous polyangiitis; SBO, skull base osteomyelitis.

**Table 4:** Univariable analysis for differentiating GPA from NPC.

Variables	OR	95% CI	*p* Value
Location			
Parapharyngeal *over* nonparapharyngeal space	7.36	1.46–37.08	0.016 *
Margin			
Well- *over* poorly defined	0.043	0.001–1.65	0.091
Partially *over* poorly defined	0.13	0.024–0.70	0.018 *
T1 signal intensity			
Isointense *over* hypointense	6.11	0.14–270.76	0.349
T2 signal intensity			
Isointense *over* hyperintense	0.09	0.003–2.56	0.159
ADC (×10^−3^ mm^2^/s)	0.97	0.02–47.69	0.986
Contrast enhancement			
Heterogeneous *over* homogeneous	1.84	0.43–7.97	0.412
Necrosis			
Present *over* absent	1.07	0.13–8.77	0.947
Extent			
Prevertebral muscle	0.25	0.028–2.16	0.207
Infratemporal fossa	0.33	0.003–32.59	0.638
Retroclival area	9.24	0.28–306.48	0.213
Cavernous sinus	2.89	0.30–27.97	0.359
Dura	26.41	3.32–209.79	0.002 *
Cranial nerve	1.85	0.43–7.97	0.412
Retropharyngeal lymphadenopathy	0.073	0.013–0.406	0.003 *

* *p* values less than 0.05 indicate statistical significance. OR, odds ratio; CI, confidence interval; GPA, granulomatous polyangiitis; NPC, nasopharyngeal carcinoma; ADC, apparent diffusion coefficient.

**Table 5:** Diagnostic performance of variables for differentiating GPA from NPC.

Variables	Sensitivity	Specificity	Positive Predictive Value	Negative Predictive Value
Parapharyngeal epicenter	66.7%	81.3%	76.9%	72.2%
Poorly defined margin	73.3%	81.3%	78.6%	76.5%
Presence of dural involvement	73.3%	93.8%	91.7%	78.9%
Absence of retropharyngeal lymphadenopathy	80.0%	18.8%	48.0%	50.0%

GPA, granulomatous polyangiitis; NPC, nasopharyngeal carcinoma.

**Table 6:** Univariable and multivariable analyses for differentiating GPA from non-GPA.

Variables	*Univariable Analysis*			*Multivariable Analysis*		
	OR	95% CI	*p* Value	OR	95% CI	*p* Value
Location						
Parapharyngeal *over* nonparapharyngeal space	6.89	1.87–25.41	0.004 *	6.26	1.63–23.97	0.008 *
Margin						
Well- *over* poorly defined	<0.001	<0.001–>999.99	0.980			
Partially *over* poorly defined	0.86	0.22–3.30	0.825			
T1 signal intensity						
Isointense *over* hypointense	>999.99	<0.001–>999.99	0.974			
T2 signal intensity						
Isointense *over* hyperintense	>999.99	<0.001–>999.99	0.976			
ADC (×10^−3^ mm^2^/s)	0.047	<0.001–4.70	0.193			
Contrast enhancement						
Heterogeneous *over* homogeneous	0.38	0.11–1.27	0.115			
Necrosis						
Present *over* absent	0.26	0.051–1.296	0.100			
Extent						
Prevertebral muscle	0.49	0.12–2.041	0.324			
Infratemporal fossa	<0.001	<0.001–>999.99	0.962			
Retroclival area	0.75	0.18–3.21	0.698			
Cavernous sinus	1.42	0.31–6.57	0.656			
Dura	3.72	1.01–13.72	0.048 *	3.21	0.79–13.04	0.103
Cranial nerve	2.31	0.68–7.89	0.183			
Retropharyngeal lymphadenopathy	0.464	0.11–1.93	0.290			

* *p* values less than 0.05 indicates statistical significance. OR, odds ratio; CI, confidence interval; GPA, granulomatous polyangiitis; NPC, nasopharyngeal carcinoma; ADC, apparent diffusion coefficient.

**Table 7:** Diagnostic performances of variables for differentiating GPA from non-GPA.

Variables	Sensitivity	Specificity	Positive Predictive Value	Negative Predictive Value
Parapharyngeal epicenter	66.7%	77.5%	52.6%	86.1%
Presence of dural involvement	73.3%	57.5%	39.2%	85.2%

GPA, granulomatous polyangiitis.

## Data Availability

Data are only available upon request, and before the request, data cannot be shared publicly, by the regulation of Institutional Review Board of Seoul National University Bundang Hospital, because data may contain potentially identifying or sensitive patient information. For researchers who may wish to have access to the data of this study, please contact the following email to send data inquiry: serenaaug@snubh.org (Research Support, Institutional Review Board of Seoul National University, Bundang Hospital).
